# Vertebral Column Resection for Kyphoscoliosis in a Patient with Ehlers-Danlos Syndrome: An Intraoperative Neurophysiological Monitoring Alert

**DOI:** 10.7759/cureus.759

**Published:** 2016-08-31

**Authors:** Faisal R Jahangiri, Sami Al Eissa, Samir Sayegh, Fahad Al Helal, Shomoukh A Al-Sharif, Monerah M Annaim, Sheryar Muhammad, Tanweer Aziz

**Affiliations:** 1 Division of Neurology, Department of Medicine, King Abdulaziz Medical City, Ministry of National Guard Health Affairs, Riyadh, Saudi Arabia; 2 Division of Orthopedics, Department of Surgery, King Abdulaziz Medical City, Ministry of National Guard Health Affairs, Riyadh, Saudi Arabia; 3 College of Medicine - Female, KSAU-HS; 4 Department of Anesthesia, King Abdulaziz Medical City, Ministry of National Guard Health Affairs, Riyadh, Saudi Arabia

**Keywords:** kyphosis, kyphoscoliosis, ehlers-danlos syndrome, intraoperative neurophysiological monitoring, ionm, ssep, mep, neurophysiology, paraplegia, o-arm

## Abstract

A 16-year-old male patient with Ehler-Danlos syndrome (EDS) and a back deformity since birth presented with severe kyphoscoliosis. The patient was neurologically intact but had respiratory and cardiac insufficiencies. A two-stage vertebral column resection (VCR) at T9-T10 with multiple level fusion with multimodality intraoperative neurophysiological monitoring (IONM) was planned.

During the first stage, pedicle screws were placed at multiple spinal levels above and below the VCR level. Upper and lower somatosensory evoked potentials (SSEP), transcranial electrical motor evoked potentials (TCeMEP), and electromyography were monitored continuously and showed no significant changes. The second stage was performed one week later. Baseline SSEP and TCeMEP responses were present in all extremities. The surgeon was informed of a sudden 70% amplitude drop in TCeMEP in the lower limbs with stable SSEP after ligating one of the left nerves/vessels fully stretching the spinal cord. The surgeon removed the ligation, and an improvement in motor responses followed. Surgery proceeded with the highest levels of caution. Later, there was a sudden loss of TCeMEP and SSEP in the lower limbs bilaterally. The correction was released, mean arterial pressure was increased, and intravenous dexamethasone was administered. The surgical correction was aborted, and the decision was made to close the site. Lower SSEP and TCeMEP responses remained absent until closing, while upper SSEP and TCeMEP responses remained stable.

A wake-up test was done after closing. The patient moved his upper limbs but was unable to move his lower limbs bilaterally. The patient was sent for a magnetic resonance imaging scan while intubated and then sent to the intensive care unit. At 24 hours and 36 hours post-operation, the patient had no sensory and motor function below the T8 level. Forty-eight hours post-operation, the patient started to feel sensory stimuli at the T10 level. At one week post-operation, the patient regained sphincter functions, and at four weeks postoperatively, the patient’s hip flexors started to recover.

VCR in patients with EDS has a very high risk of damaging the spinal cord due to the fragile vasculature of the spinal cord. Real-time IONM is useful in the early identification of spinal cord injury in cases of this nature.

## Introduction

Kyphosis describes the curvature of the thoracic spine; this term is used to describe the normal and abnormal curvature. Hyperkyphosis is a better way to describe an excessive thoracic curvature. Kyphosis can occur due to postural or structural cases. Types of kyphosis depend on the age of the patient and the cause of the condition. Congenital kyphosis is more often seen in babies and children due to vertebral anomalies and is subdivided into Type 1 (resulting from a failure of formation of vertebral bodies) and Type 2 (resulting from a failure of segmentation of vertebral bodies). Scheuermann's disease (or juvenile osteochondrosis) mainly affects adolescents and is usually characterized by a rigid kyphosis and wedged thoracic vertebrae. Patients of kyphosis present with back pain, back hump, and fatigue. In severe cases, it can affect a patient’s breathing and cause nerve compression and lumbar lordosis [1].

Kyphosis is diagnosed clinically by assessing spine curvature, the range of motion, and examination of the nervous system. A lateral x-ray is used to assess the degree of curvature and findings in each type of kyphosis (e.g., Schmorl's nodes in Scheuermann's disease). Other diagnostic tools for determining kyphosis include magnetic resonance imaging (MRI), bone density tests (in elderly patients), pulmonary function tests, and nerve evaluations. The management of kyphosis depends on the degree of spinal curvature and the specific type of kyphosis. Postural kyphosis is usually treated with postural correction and exercises. Other types of kyphosis are treated based on the degree of curvature, the age of the patient, and the underlying cause. For example, Scheuermann's disease is treated with conservative methods, such as bracing, nonsteroidal anti-inflammatory drugs, and physiotherapy if the spinal curvature is less than 60 degrees. Congenital kyphosis is usually treated surgically.

Ehlers-Danlos syndrome (EDS) is a group of rare genetic connective tissue disorders characterized by various manifestations in the skin, joints, and connective tissues. The frequency of EDS is 1 in 5,000 [2]. The most common type of EDS is hypermobile EDS. The diagnosis is usually based on family history and clinical criteria (e.g., clinical condition of the vasculature, skin, joints, and skeleton). In some patients, genetic testing may be useful for an EDS diagnosis.

Intraoperative neurophysiological monitoring (IONM) has been utilized in scoliosis surgeries for over three decades. To maximize the benefit of IONM, a multimodality approach is recommended that includes somatosensory evoked potentials (SSEPs), transcranial electrical motor evoked potentials (TCeMEPs), and spontaneous and triggered electromyography (s-EMG and t-EMG) [3]. TCeMEPs and SSEPs are reliable in detecting any spinal cord changes during scoliosis and kyphoscoliosis surgeries [4]. A multimodality approach can minimize any postoperative neurological deficits due to surgical manipulation and correction. Real-time monitoring also provides quick feedback to the operating surgeon to help avoid any permanent ischemic or neurological changes [5].

Vertebral column resection (VCR) for kyphoscoliosis has a high risk of damaging the motor and sensory pathways due to their proximity to the spinal cord and nerve roots [6]. Using TCeMEP and SSEP during the resection and correction can aid in both early detection and minimizing neural injuries. This case report examines the benefits of IONM use during the surgical treatment of kyphoscoliosis in a patient with EDS.

## Case presentation

### Patient history 

A 16-year-old male patient with Ehler-Danlos syndrome and a back deformity since birth presented with severe kyphoscoliosis. The patient was neurologically intact but had respiratory and cardiac insufficiencies. Informed patient consent was obtained from his parents for treatment.

**Figure 1:**
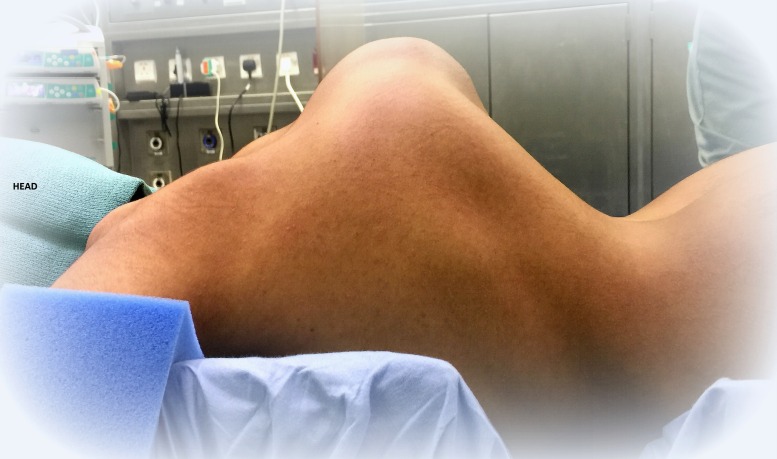
Stage 1: Patient in the prone position after intubation.

A two-stage VCR at T9-T10 with multiple-level fusion was planned (Figure 1). After intubation, electrodes were placed for upper and lower SSEP, TCeMEP, and EMGs. Baseline SSEP and TCeMEP responses were present in all limbs. 

### Anesthesia

Propofol (120 to 140 mcg/kg/minute) and remifentanil (0.1 mcg/kg/minute) infusions comprised the total intravenous anesthesia used. At intubation, a neuromuscular blocking agent with a very short duration was used [7]. A train of four (TOF) monitoring technique was utilized by stimulating the posterior tibial nerves and recording from the corresponding extensor hallucis brevis muscles. A TOF of 4/4 was maintained during the entire surgical procedure.

### Intraoperative neurophysiological monitoring

IONM (including SSEP, TCeMEP, and EMG) was performed during this two-stage surgical procedure. After the intubation, surface adhesive stimulation electrodes were placed bilaterally on the ulnar nerves at the wrist and the posterior tibial nerves at the ankles for eliciting SSEPs [7]. Baseline SSEPs were recorded in both the upper and lower extremities (ulnar: stimulation intensity - 22 mA, duration - 300 µsec, repetition rate - 3.66 Hz; posterior tibial: stimulation intensity - 60 mA, duration - 300 µsec, repetition rate - 3.66 Hz). Subdermal recording needle electrodes were placed for SSEPs at C3´ (at CP3), C4´ (at CP4), Cz´ (at CPz), CV5 (at the fifth cervical spine vertebra), Fpz and Erb (placed at left and right Erb points) (Figure 4). A 32-channel Medtronic NIM-Eclipse™ neuromonitoring system (Medtronic, Inc., Minneapolis, MN, USA) was used for IONM.

Corkscrew electrodes were placed on the patient’s scalp at C1/C2 and C3/C4 for TCeMEP stimulation. Trains of seven to nine square-wave stimuli with 75-µsec durations and intensities ranging from 150 to 400 volts were utilized. EMG and TCeMEP recordings were performed by placing subdermal needle electrodes in the thenar and hypothenar muscles in the hand, the quadriceps, tibialis anterior, gastrocnemius, abductor hallucis, and extensor hallucis brevis muscles in the lower extremities. 

### Surgical procedure

Stage I

**Figure 2:**
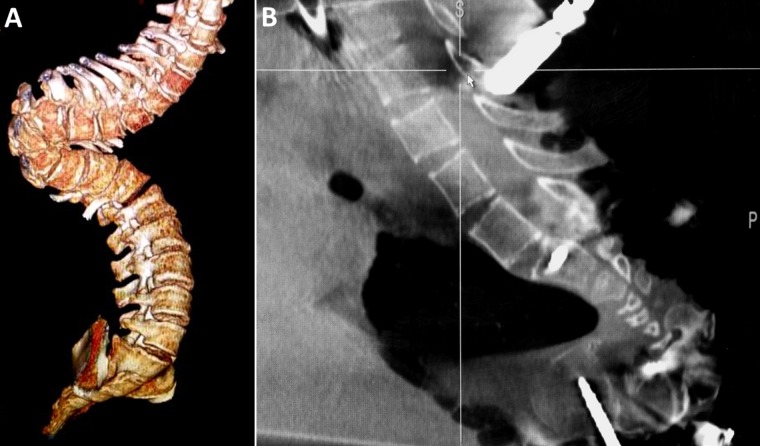
Stage 1: A) 3-D image of the spine. (B) Intraoperative O-Arm image of the spine showing the kyphoscoliosis.

**Figure 3:**
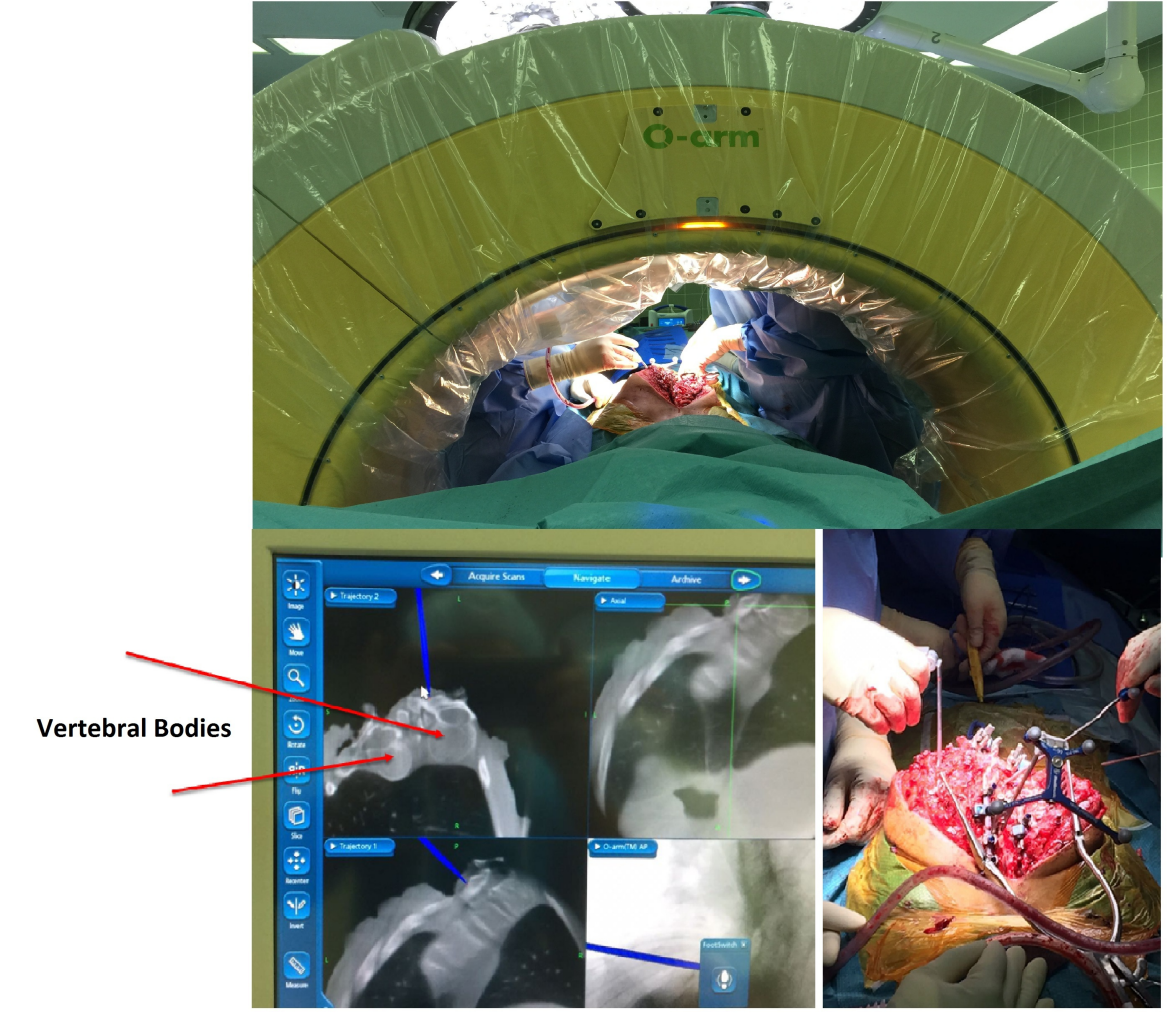
Stage 1: O-Arm in use for placement of pedicle screws.

**Figure 4:**
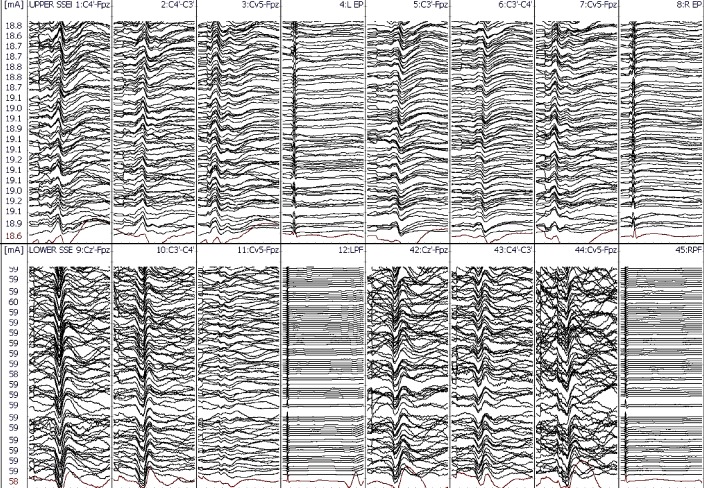
Stage 1: Upper (ulnar) and lower (posterior tibial nerve) extremities somatosensory-evoked potentials (SSEP) during the first stage. No changes in SSEP responses.

**Figure 5:**
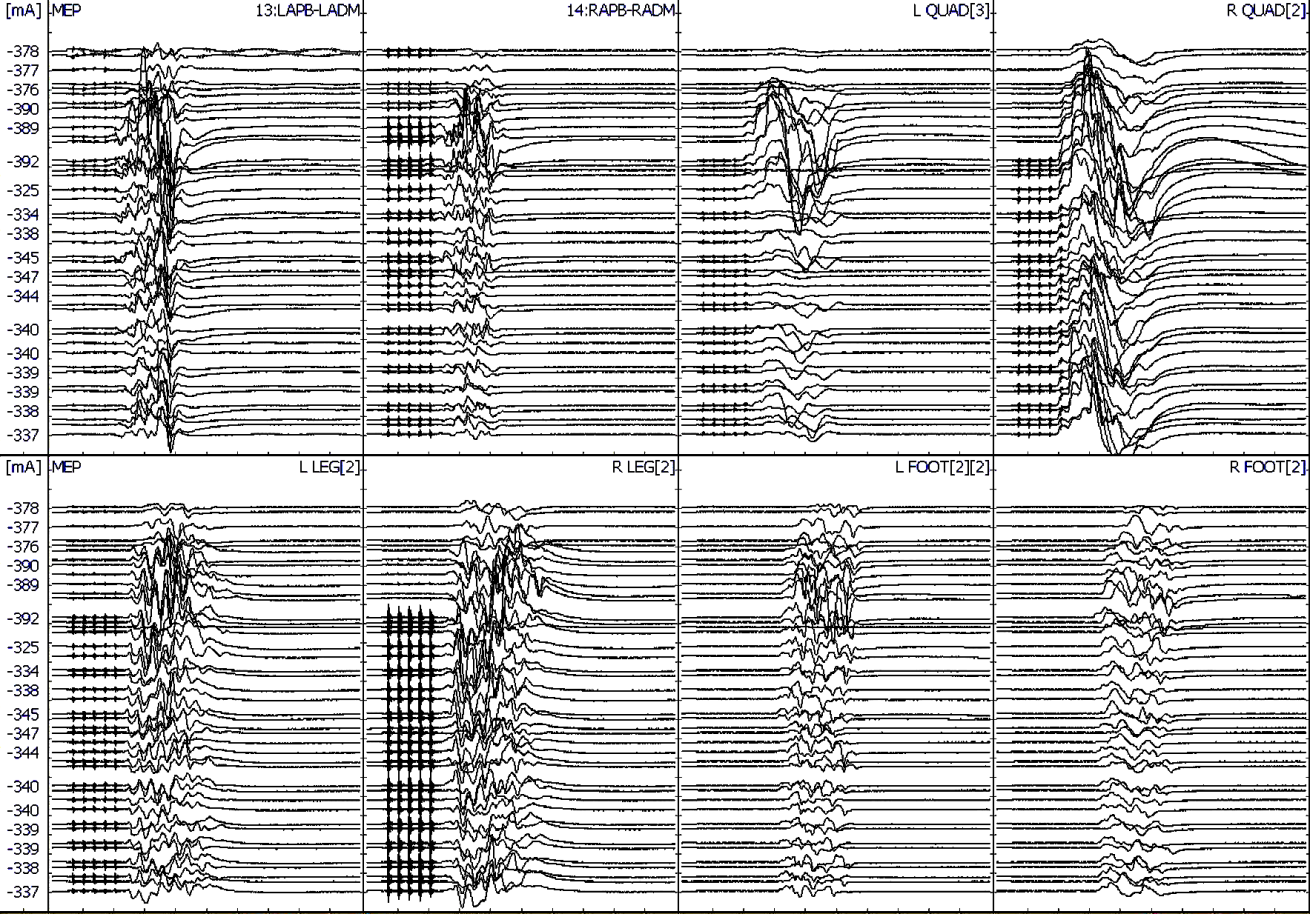
Stage 1: Upper and lower extremities transcranial electrical motor-evoked potentials (TCeMEP) during the first stage. No changes in TCeMEP responses.

During the first stage, only pedicle screws were placed at multiple spinal levels above and below the VCR level (Figure 2). Medtronic’s O-arm navigation system was used for placing thoracic pedicle screws (Figure 3). Upper and lower SSEPs, TCeMEPs, and EMGs were monitored continuously, and no significant changes were noted (Figures 4-5).

Stage II 

**Figure 6:**
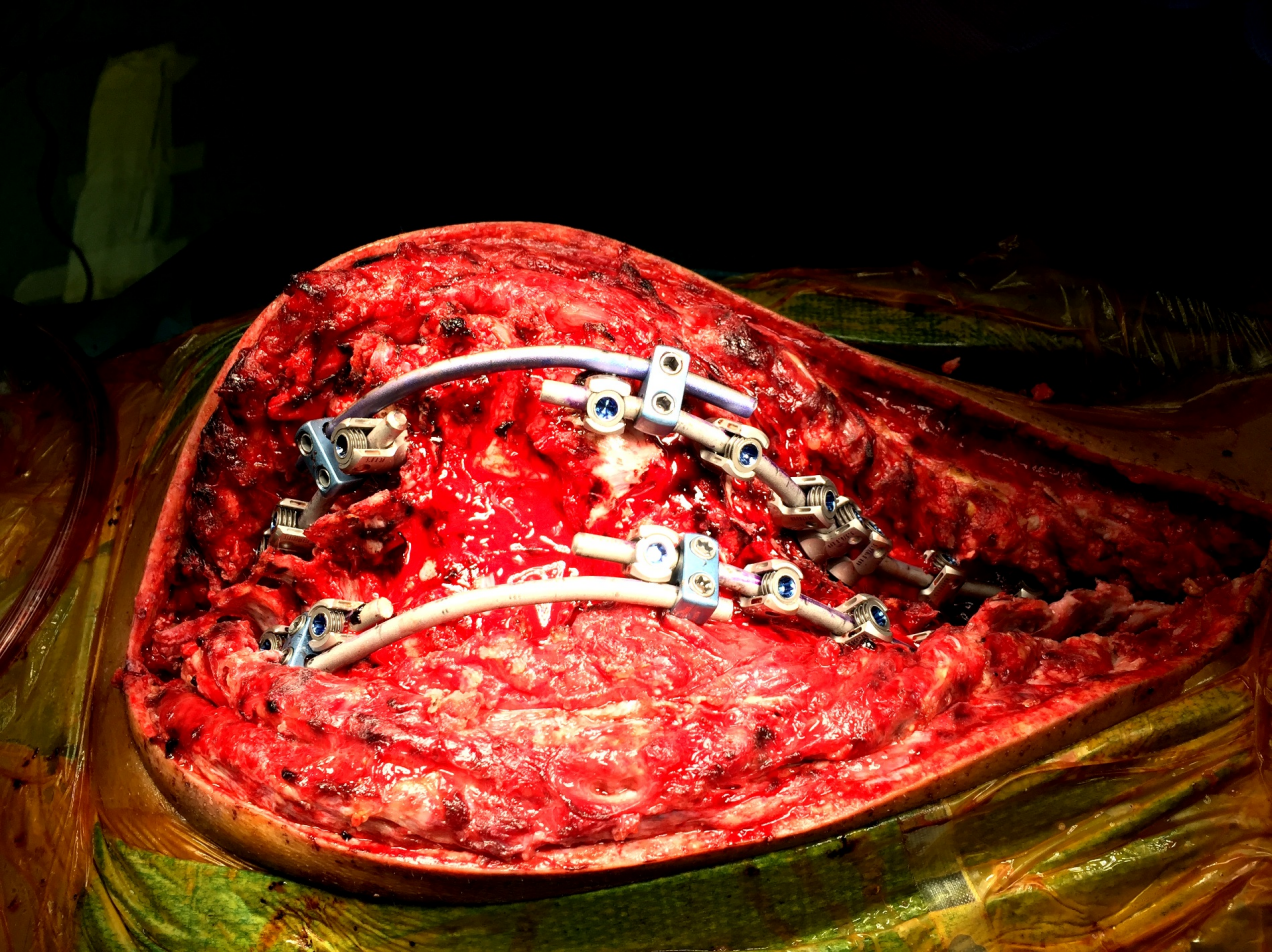
Stage 2: Intraoperative instrumented fusion of the spine.

**Figure 7:**
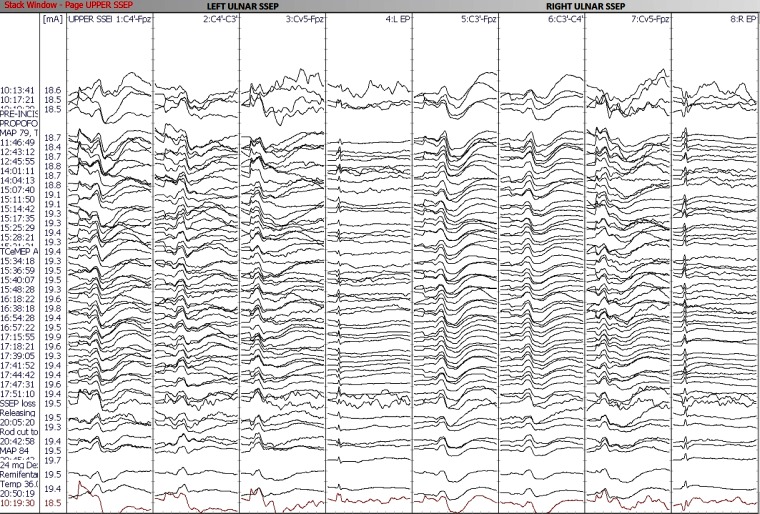
Stage 2: No changes in upper extremity (ulnar nerve) SSEP responses.

**Figure 8:**
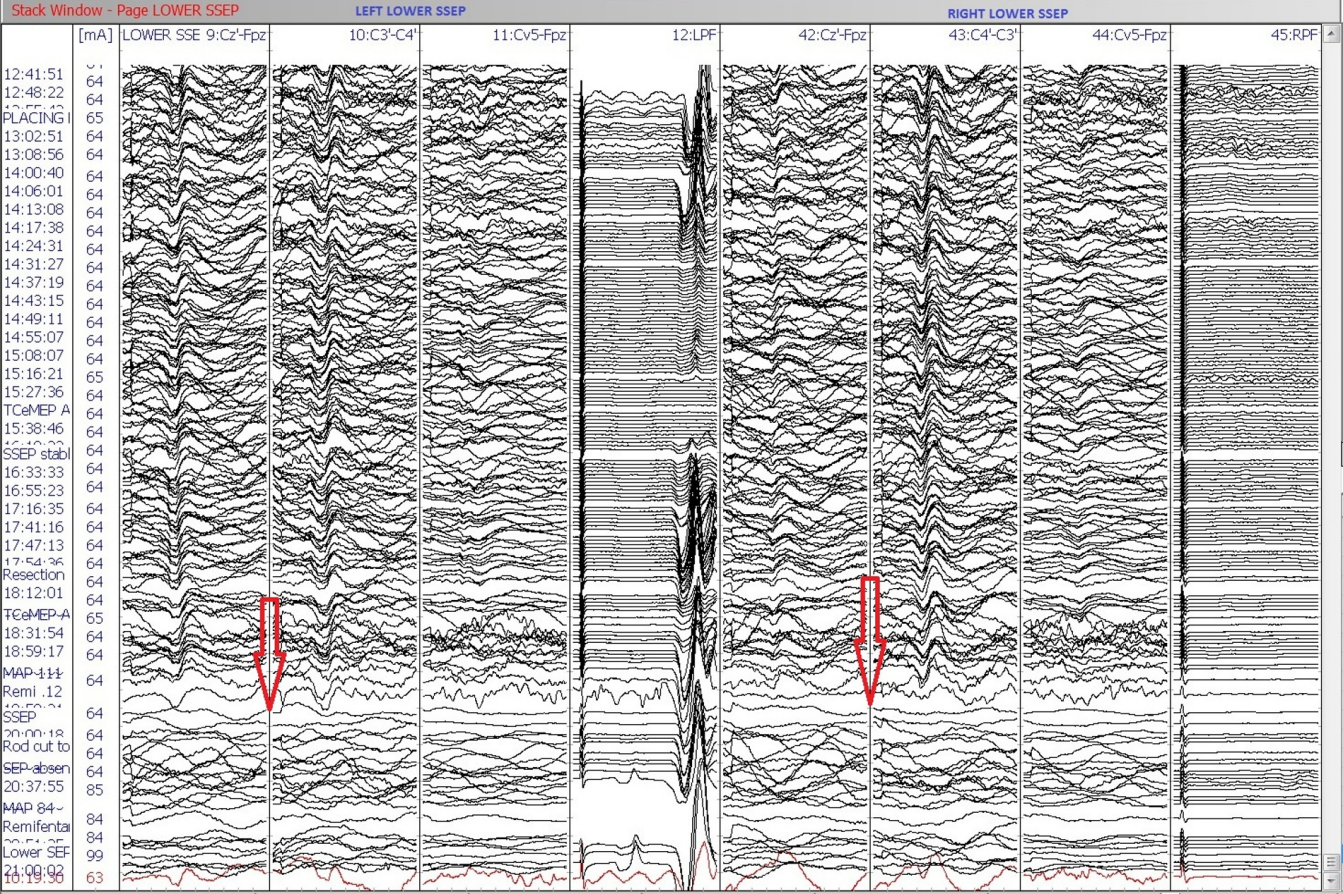
Stage 2: Sudden loss of bilateral lower extremity (posterior tibial nerve) SSEP responses.

**Figure 9:**
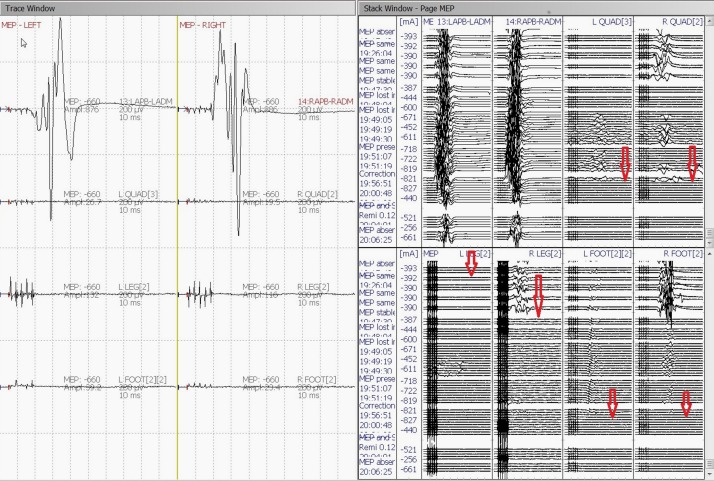
Stage 2: Loss of bilateral lower extremity motor-evoked potentials (TCeMEP) responses. Left: Average TCeMEP responses showing responses present in hand muscles with loss of responses in lower extremities. Right: Stack TCeMEP responses showing loss of lower extremity muscles responses.

The second stage was performed one week later, for which a VCR at T9-T10 was planned (Figure 6). At post-intubation, SSEP and TCeMEP responses were present in all four extremities. Surgery was started at 08:00. At 15:49, the surgeon was informed of a sudden drop in TCeMEP response in the lower limbs after ligating one of the left nerves/vessels, fully stretching the spinal cord. The surgeon removed the ligation and an improvement in motor responses followed. The surgeon was informed of a 70% amplitude drop in TCeMEP at 18:19 in both lower limbs, with stable SSEP (Figure 7). Surgery proceeded with the highest level of caution. There was a sudden loss of SSEP and TCeMEP in the lower limbs bilaterally at 19:59 (Figures 8-9). The correction was released, mean arterial pressures were increased to over 100 mm Hg, and 24 mg of dexamethasone was administered intravenously. Surgical correction was aborted and the surgical site was closed. Lower SSEP and TCeMEP responses remained absent until closing, while the upper SSEP and TCeMEP remained stable.

### Postoperative 

**Figure 10:**
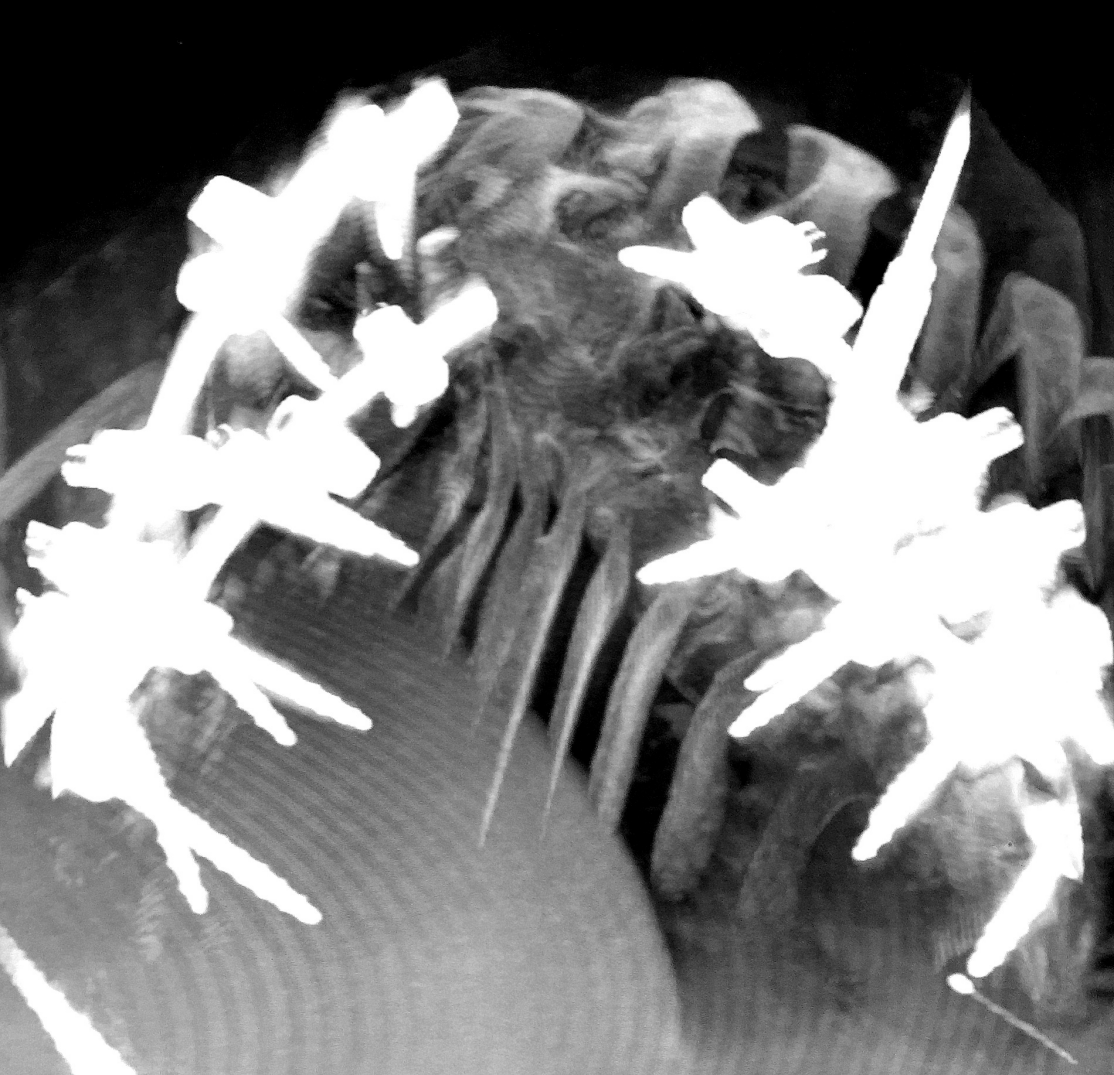
Stage 2: Postoperative O-Arm image of the spine showing the instrumented fusion.

A wake-up test was done after closing. The patient moved his upper limbs but was unable to move his lower limbs bilaterally (Figure 10). The patient was sent for an MRI while intubated and then sent to the intensive care unit (ICU). Twenty-four hours and 36 hours postoperatively, the patient had no sensory and motor function below the level of T8. Forty-eight hours postoperatively, the patient started to feel sensory stimuli at the T10 level. One week postoperatively, the patient regained sphincter functions. Four weeks postoperatively, the patient’s hip flexors started to recover.

## Discussion

Type IV EDS (i.e., vascular EDS) is a potentially life-threatening disease. There is an increased risk of spontaneous vascular or visceral rupture of large arteries in these patients. Patients with a different kind of EDS, Type VI EDS (i.e., kyphoscoliosis EDS), need surgical intervention to treat respiratory complications due to progressive kyphoscoliosis [8]. A spontaneous vascular rupture may also result in some of these patients due to fragile vascular structures. Kyphoscoliosis is treated by an orthopedic surgeon and may require braces and physical therapy, in addition to the surgery. Due to a high mortality rate and complications in patients with EDS, great attention should be paid during the surgical procedures of these patients. Surgeons should be aware of the vascular complications to avoid intraoperative vascular insults that may lead to spinal cord ischemia and postoperative neurological deficits. The surgical correction of kyphoscoliosis in patients with Ehlers-Danlos syndrome has very high risk of paraplegia and other neurological deficits [9-10]. Patients with EDS have very fragile vasculature as well as joint mobility limitations. Therefore, a vertebral column resection has a very high risk of damaging the spinal cord in a kyphoscoliosis patient with EDS due to the removal of the bone tissue protecting the spinal cord. Patient selection for surgical intervention should be done very carefully, given the high risk of paralysis. 

## Conclusions

A vertebral column resection in patients with Ehler-Danlos syndrome carries a very high risk of damaging the spinal cord due to vascular abnormalities. In our case, real-time IONM proved useful for the early identification of spinal cord injury during the surgical procedure. During surgery, our patient lost his sensory and motor functions below the level of T8. Due to the continuous neuromonitoring of TCeMEP and SSEP, the surgery was aborted in a timely manner, thus minimizing the duration of spinal ischemia and allowing for an improved postoperative recovery for the patient. In order to minimize postoperative neurological deficits, we highly recommend utilizing continuous TCeMEP and SSEP monitoring during VCR and pedicle screw placement for spinal correction procedures to assist with the prevention of injury to the spinal cord for patients with EDS. 
